# Incident Angle- and Polarization-Insensitive Metamaterial Absorber using Circular Sectors

**DOI:** 10.1038/srep27155

**Published:** 2016-06-03

**Authors:** Dongju Lee, Jung Gyu Hwang, Daecheon Lim, Tadayoshi Hara, Sungjoon Lim

**Affiliations:** 1 School of Electrical and Electronic Engineering, Chung-Ang University, Heukseok-Dong, Dongjak-Gu 156-756, Republic of Korea

## Abstract

In this paper, an incident angle- and polarization-insensitive metamaterial absorber is proposed for X-band applications. A unit cell of the proposed absorber has a square patch at the centre and four circular sectors are rotated around the square patch. The vertically and horizontally symmetric structure of the unit cell enables polarization-insensitivity. The circular sector of the unit cell enables an angle-insensitivity. The performances of the proposed absorber are demonstrated with a full-wave simulation and measurements. The angular sensitivity is studied at different inner angles of the circular sector. When the inner angle of the circular sector is 90°, the simulated absorptivity is higher than 90%, and the frequency variation is less than 0.96% for incident angles up to 70°. The measured absorptivity at 10.44 GHz is close to 100% for all the polarization angles under normal incidence. When the incident angles are varied from 0°– 60°, the measured absorptivity is maintained above 90% for both the transverse electric (TE) and the transverse magnetic (TM) modes.

Electromagnetic (EM) metamaterials (MMs) are artificial materials engineered to have unique properties that are not found in nature^1^. For instance, MMs can manipulate the effective permittivity or permeability. MMs can be implemented by a periodic array of sub-wavelength resonators such as the split-ring resonators (SRR)^2^. Owing to their extraordinary properties, various applications of MMs like super lenses^3^, miniaturized microwave components^4^, and MM absorbers^5^ have been reported.

In particular, MM absorbers have been studied since Landy introduced them in 2008^2^. They generate electric and magnetic resonances that are independently manipulated for effective permittivity and permeability, respectively. MM absorbers have certain advantages compared to conventional absorbers like ferrite^6^, wedged-tapered absorbers^7^ and Salisbury screen absorbers^8^. The MM absorber can achieve a high absorptivity in spite of a thin substrate. In addition, functional absorbers can be realized with tunable devices or materials. Because of these advantages, the MM absorber has been researched for various applications of the spectrum, from microwave to optical signals. As an MM absorber is based on a periodic array of resonators, it operates at a specific frequency and has a narrow-bandwidth. Studies on increasing the bandwidth of the MM absorbers have been reported^9^,^10^,^11^. In general, absorptivity of an MM absorber is also dependent on the incident angle and the polarization. Polarization-insensitive MM absorbers can be achieved by designing a horizontally and vertically symmetric unit cell^12^,^13^. Several polarization- and angle-insensitive MM absorbers have been presented^14^,^15^,^16^.

In this work, a novel angle- and polarization-insensitive metamaterial absorber is proposed for X-band applications. The unit cell of the proposed absorber has a square patch at the centre and four circular sectors are rotated around the square patch. The vertically and horizontally symmetric structure of the unit cell enables polarization-insensitivity. The circular sector of the unit cell enables angle-insensitivity as demonstrated by the parametric study of the absorptivity at different inner angles of the circular sector. The performances of the proposed absorber are demonstrated with a full-wave simulation and measurements.

## Results

### Absorber design and fabrication

**Figure 1:**
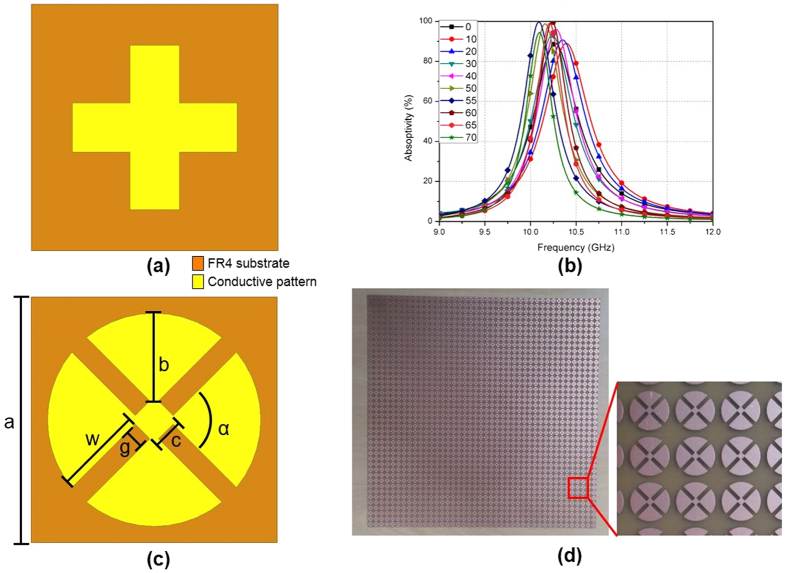
(**a**) Primitive JC unit cell. (**b**) Simulated absorptivity of the JC unit cell at different incident angles. **(c)** The proposed unit cell with circular sectors: *a* = 6 mm, *b* = 2.2 mm, *c* = 0.8 mm, g = 0.5 mm, w = 2.3 mm and α = 90° (**d**) Fabricated absorber prototype.

**Figure 2:**
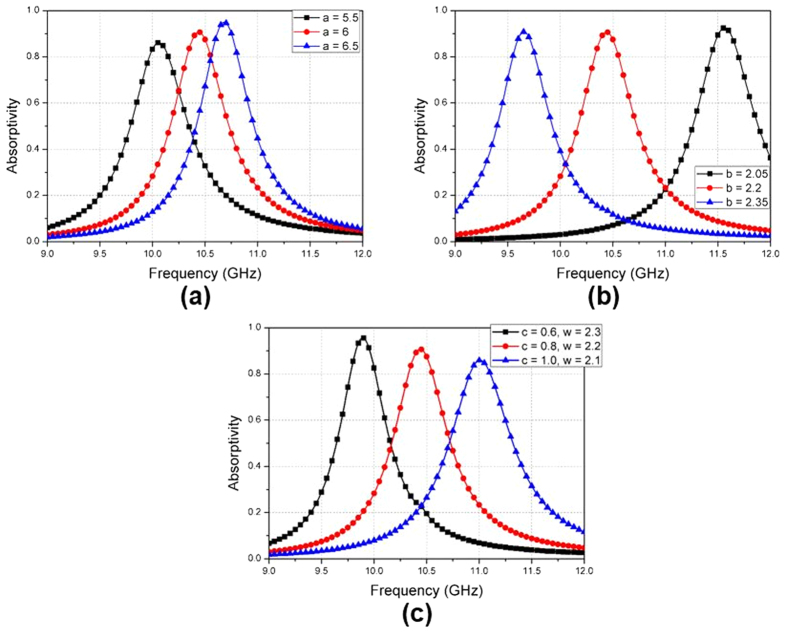
Simulated absorptivity of the proposed unit cell at different values of the parameters: (**a**) When *a* varies from 5.5 mm–6.5 mm (**b**) When *b* varies from 2.05 mm–2.35mm (**c**) When *c* varies from 0.6 mm–1 mm which corresponds the change of w from 2.3 mm–2.1 mm.

Figure 1 shows the geometry of the proposed unit cell and the fabricated prototype absorber sample. For generating electric and magnetic resonances, a bi-layered conductive structure is necessary. The top surface of the unit cell contains a square patch at the centre and there are four circular sectors around the square patch. The bottom surface of the unit cell is completely covered with a conductor. The conductive patterns on the top and bottom surfaces are realized by copper. The proposed unit cell in Fig. 1(b) is initiated from a well-known Jerusalem Cross (JC) resonator, depicted in Fig. 1(a)^17^. Because of their symmetry, both the unit cells are insensitive to polarization. However, the absorptivity of the JC unit cell in Fig. 1(a) varies for different incident angles as shown in Fig. 2. In order to make the absorptivity insensitive to the incident angles, the circular sector unit cell in Fig. 1(b) is proposed in this work.

This is demonstrated using a full-wave simulation. To experimentally demonstrate the idea presented, the proposed MM absorber with 42 × 42 unit cells is fabricated on a low-cost FR4 substrate, as show in Fig. 1(c). The FR4 substrate has a relative permittivity of 3.9, a dielectric loss of 0.02, and a thickness of 0.6 mm. The overall size of the prototype absorber is 252 mm × 252 mm.

When EM waves are incident on the MM absorber, there are reflected and transmitted waves owing to discontinuity of the boundary. The absorptivity (A) can be calculated from the reflection coefficient (Γ) and the transmission coefficient (T), as follows:


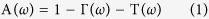


Therefore, perfect absorptivity can be achieved when the reflection and transmission coefficients are zero.

Under normal incidence, the reflection coefficient is given by,


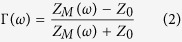


where Z_M_ (*ω*) and Z_0_ are the impedances of the MM absorber and the free space, respectively. From Eq. (2), it can be seen that the reflection coefficient becomes zero when Z_M_ (*ω*) and Z_0_ are matched. Z_M_ (*ω*) can be controlled by tailoring the effective permittivity (*ε*_M_) and the permeability (*μ*_M_) as follows^18^:


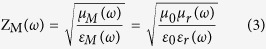


where *ε*_0_ and *μ*_0_ are the permittivity and the permeability of free space, respectively. *ε*_r_ and *μ*_r_ are the relative permittivity and permeability, respectively. After impedance matching between the MM absorber and free space, the transmission coefficient can be minimized by dissipating the transmitted wave with significant dielectric losses. The loss factor of the MM absorber is high because of the large imaginary parts of the refractive index (n). Therefore, a high absorptivity can be achieved by designing an MM absorber with zero reflection and transmission coefficients.

However, the zero reflection condition changes for an oblique incidence because the reflection coefficients for perpendicular polarization (Γ_⊥_) and parallel polarization (Γ_||_) are given by,


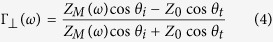



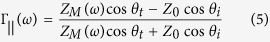


Although the MM absorber shows perfect absorptivity under normal incidence, its absorptivity changes when the incident angles are varied as per Eqs. (2), (4), and (5). Therefore, an angle-insensitive unit cell must be designed for obtaining an angle-insensitive MM absorber.

### Simulated and experimental results

For designing the proposed unit cell as shown in Fig. 1(c), five metric parameters (*a*, *b*, *c, g, w*) and one angular parameter *α*, are determined using full-wave simulation. *a* indicates the length of a side of the unit cell; *b* is the radius of a circular sector; *c* represents the length of the square patch at the centre; g is the gap spacing between each circular sector; w represents the gap width between each circular sector; α is the inner angle of a circular sector. Figure 2 shows the simulated absorptivity at different values of the *a*, *b*, and *c* parameters. When *a* increases, the resonant frequency increases and the absorptivity slightly increases as shown in Fig. 2(a). It is observed from Fig. 2(b) that the resonant frequency decreases with a larger value of b as shown in Fig. 2(b). The resonant frequency can be calculated from effective inductance (L) and effective capacitance (C)^19^,^20^. When *b* is larger, effective inductance is increased so that the resonant frequency decrease from *f* = 1/√LC. When square patch size (c) is larger, gap width (w) becomes narrower because other parameters (a, b, g) are fixed. Therefore, effective capacitance is decreased with larger c (smaller w)^20^. Therefore, the resonant frequency increases with larger c because of *f* = 1/√LC.

The frequency variations are related to the changes in the effective resonance wavelength^19^. Therefore, the resonant frequency decreases with a larger value of b as shown in Fig. 2(b). However, the resonant frequency increases as larger value of c (larger square patch size). Therefore, it is better to understand this in terms of effective capacitance (permittivity) and inductance (permeability)^21^. When c is larger, the gap width (w) is decreased as well. Therefore, the resonant frequency increases because effective capacitance is decreased. Similarly, when b is larger, effective inductance and permeability are increased. Therefore, the resonant frequency decrease with larger b. Therefore, the three metric parameters *a*, *b*, and *c* determine the resonant frequency of the proposed MM absorber.

**Figure 3:**
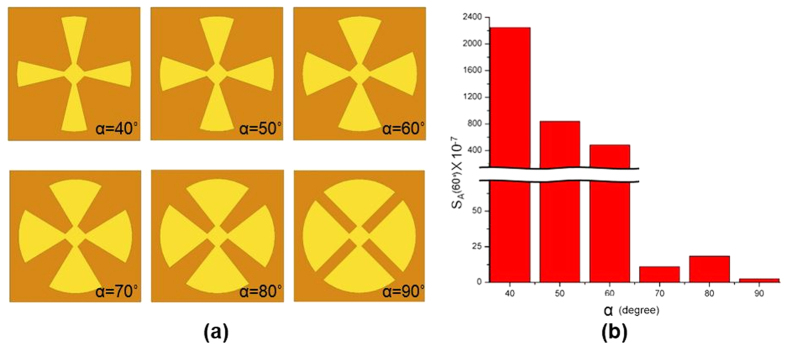
(**a**) Six unit cells with circular sectors at α = 40°, 50°, 60°, 70°, 80°, and 90°, (**b**) Angle-sensitivity (S_A_ (θ)) of the six unit cells when the incident angle (θ) is 60°.

The circular sector is proposed for angle-insensitivity, in this study. The inner angle (*α*), especially, affects the angle-insensitivity. To demonstrate this, six unit cells with different values of *α*: (40°, 50°, 60°, 70°, 80°, 90°) are designed, as shown in Fig. 3(a). Their absorptivity is simulated at incident angles (*θ*) of 0° and 60°. To present the relationship between *α* and the angle-sensitivity, the angle-sensitivity (S_A_) is defined as


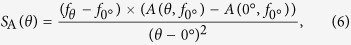


where *f*_θ_ and *f*_0_° are the resonant frequencies at *θ* and at normal incidence, respectively. A(*θ*, *f*_θ_) is the absorptivity at an incident angle of *θ* and a frequency of *f*_θ_. A(*θ*, *f*_0_°) is the absorptivity at an incident angle of *θ* and a frequency of *f*_0_°. Figure 3(b) shows the S_A_ (60°) when *α* is varied from 40°–90°. When *α* is 90°, *f*_60_° and *f*_0_° are 10.45 GHz and 10.44 GHz, respectively. In addition, A (60°, *f*_0_°) and A (0°, *f*_0_°) are 0.99 and 0.91, respectively. Therefore, the lowest angle-sensitivity is observed at *α* = 90°.

**Figure 4:**
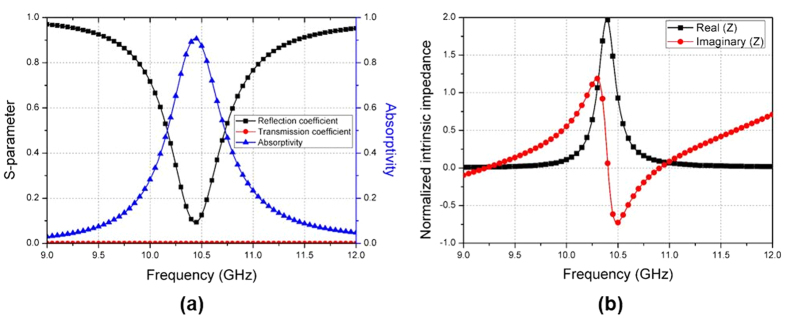
(**a**) Simulated reflection coefficient, transmission coefficient, and absorptivity of the proposed MM absorber (**b**) Normalized complex impedance of the proposed MM absorber.

Figure 4 shows the simulated absorptivity and the complex intrinsic impedance of the proposed absorber normalized to the impedance of free space under normal incidence. The complex impedance (**Z**) can be calculated from S-parameter (**S**)^22^.


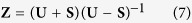


where **U** is an identify matrix.

It is observed from Fig. 4(a) that the proposed MM absorber shows 91% absorptivity at 10.44 GHz. The absorptivity is not 100% because the real part of the normalized impedance is higher than one. Although absorptivity can be 100% under normal incidence, the unit cell is designed to have higher than 90% absorptivity for incident angles from 0°–70°.

**Figure 5:**
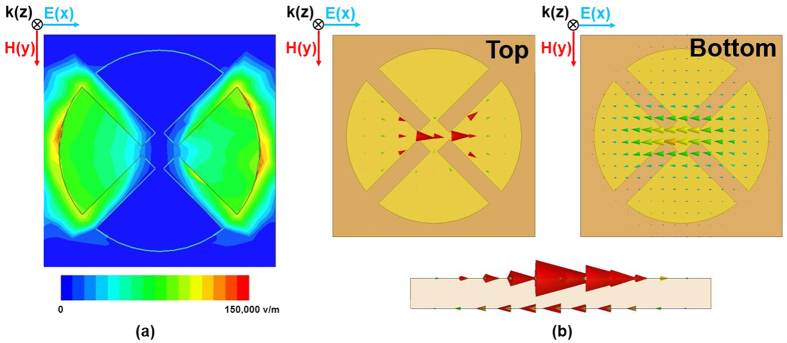
(**a**) Magnitude of the electric-field distribution (**b**) Top, bottom, and side views of the vector electric current distributions.

In order to view the electric and magnetic resonances, the magnitude of the electric field and the vector current distributions at 10.44 GHz are plotted in Fig. 5. It is observed in Fig. 5(a) that the electric fields are concentrated in the edges of the circular sector. Magnetic resonance from the surface current densities is observed in Fig. 5(b), where the surface currents excited in the two metal layers are anti-parallel.

**Figure 6:**
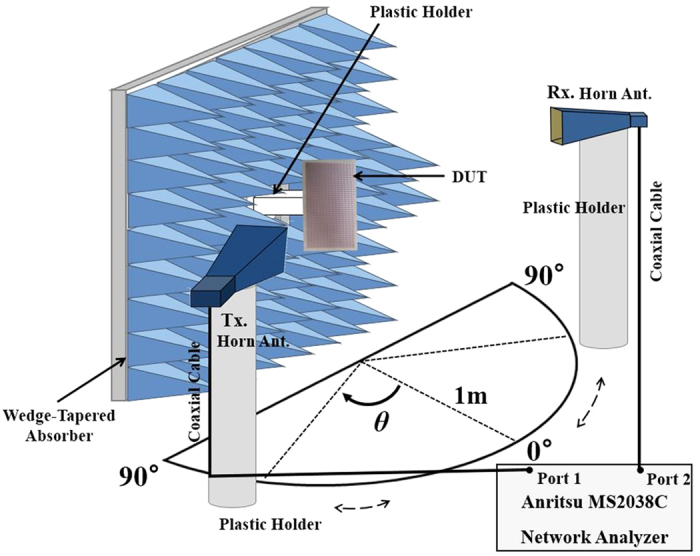
Test setup to measure the absorptivity of the fabricated MM absorber at different polarizations (φ) and incident angles (θ).

**Figure 7:**
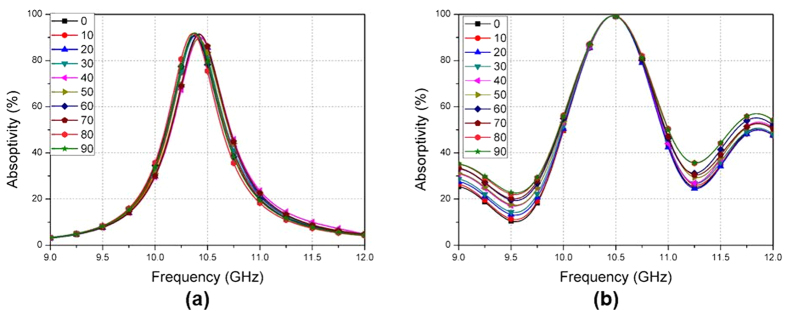
(**a**) Simulated and (**b**) Measured absorptivity of the proposed MM absorber for different polarization angles (φ) from 0°–90°.

The designed MM absorber is fabricated with 42 × 42 unit cells on an FR4 substrate. The absorptivity of the fabricated MM absorber depicted in Fig. 1(c) is measured in free space^23^. The test setup is illustrated in Fig. 6. For oblique incidence, two standard-gain horn antennas are used as the transmitting and receiving antennas. For normal incidence, a single antenna is enough to measure reflection coefficient. Antennas are located 1 m away from the absorber sample to satisfy a far-field condition. The S-parameters are measured by an Anritsu MS2038C vector network analyser (VNA) and the absorptivity is calculated from the S-parameters. To measure the wave reflected only from the fabricated absorber sample, wedge-tapered absorbing materials are used to surround the absorber sample, as illustrated in Fig. 6 and the time-gaiting function of the VNA is used. Under normal incidence, the absorptivity of the proposed MM absorber is measured at different polarization angles (φ). Figure 7 shows the simulated and measured absorptivity of the proposed MM absorber when φ is varied from 0°–90°. The measured absorptivity is almost 100% at 10.44 GHz for all polarizations. The simulated and measured absorption frequencies are identical. The measured absorptivity is higher than the simulated absorptivity because of the higher dielectric losses of the FR4 substrate.

Next, the absorptivity of the fabricated absorber is measured at different incident angles (θ). The transmitting horn antenna is rotated from 0°–70°. At each incident angle, the receiving horn antenna is located at an angle to satisfy Snell’s law and the measured S-parameters. Because the reflection coefficients for the TE and TM modes are different, the absorptivity at oblique incidence is measured for both the TE and the TM modes.

**Figure 8:**
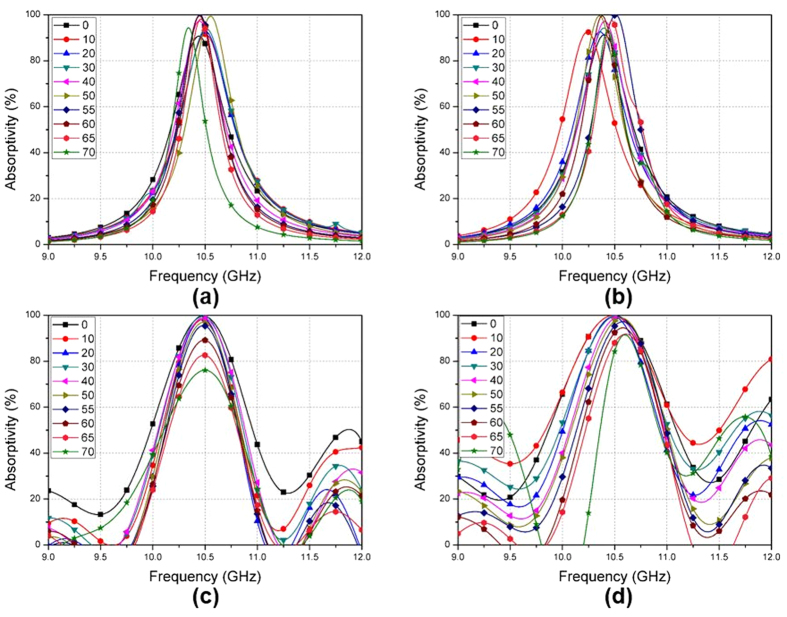
Absorptivity of the proposed MM absorber at different incident angles (θ) from 0°–70°. Simulated results in (**a**) The TE mode and (**b**) The TM mode. Measured results in (**c**) The TE mode and (**d**) The TM mode.

Figure 8 shows the simulated and measured absorptivity of the proposed MM absorber at different incident angles (θ). When θ is varied from 0°–70°, the simulated absorptivity for the TE and TM modes are plotted in Fig. 8(a,b), respectively. The simulated absorptivity is higher than 90% and the frequency variation is less than 0.96% for incident angles up to 70°. It is observed in Fig. 8(c) that the measured absorptivity at 10.44 GHz for the TE mode is higher than 90% when θ is varied from 0°–60°. At an incident angle of 70°, the absorptivity decreases to 75%. The absorption frequencies are not changed from θ = 0°–70°. Figure 8(d) shows that the measured absorptivity at 10.44 GHz for the TM mode is higher than 90% when θ is varied from 0°–60°. At an incident angle of 70°, the absorptivity decreases to 80%. The variation in the absorption frequencies at θ = 0°–70°, is 0.86%. Although the measured angle-insensitivity of the fabricated absorber is slightly degraded compared to the simulation results, the proposed MM absorber achieved an absorptivity higher than 90% for incident angles up to 60°.

## Discussion

In this paper, an angle- and polarization-insensitive MM absorber is proposed. The unit cell of the proposed MM absorber consists of four circular sectors and a square on the top layer. The circular sector, in particular, contributes to the angle-insensitivity of the MM absorber and its parameters are optimized for the lowest angle-sensitivity. The proposed absorber sample is fabricated with 42 × 42 unit cells on an FR4 substrate. The absorptivity of the fabricated sample is measured at different polarization and incident angles. Under normal incidence, the fabricated MM absorber exhibits an almost perfect absorptivity at 10.44 GHz for all polarization angles. For oblique incidence, the simulated absorptivity is higher than 90% and the frequency variation is less than 0.96% for incident angles up to 70°. The measured absorptivity is higher than 90% for incident angles up to 60°. Therefore, the polarization- and angle-insensitivity of the proposed MM absorber is successfully demonstrated using a full-wave simulation and measurements.

## Methods

### Measurement

Absorptivity of the fabricated absorber was calculated from the S-parameters that are measured by the Anritsu MS2038C vector network analyser (VNA). Two standard- gain horn antennas were used as the transmitting and receiving antennas, and each antenna was connected to the 2-port VNA. They were located 1 m away from the absorber sample to satisfy the far-field condition. The fabricated absorber sample was placed in the centre of a board surrounded by wedge-tapered absorbers to prevent unwanted reflection signals. In addition, the time gating method in the VNA was applied to measure the wave reflected only from the absorber sample. Before measuring the reflection coefficients of the proposed absorber, we calibrated the reflection coefficient of a copper plate as -1. The size of the copper plate size is same as that of the absorber. To demonstrate the polarization sensitivity, the absorptivity of the fabricated absorber was measured when the absorber sample was rotated from φ = 0°–90°. The transmitting antenna was fixed at θ = 0° for normal incidence. To demonstrate the angle sensitivity, the transmitting antenna was rotated from θ = 0°–70°. At each incident angle, the absorptivity of the fabricated absorber was measured with the receiving horn antenna that was located at an angle to satisfy Snell’s law.

### Simulation

To design the unit cell of the proposed MM absorber, a finite element method (FEM)-based ANSYS high-frequency structure simulator (HFSS) is used. For an infinite periodic structure, master/slave boundary conditions and Floquet port excitations are used in the simulation setup. The electric field and current distributions of the unit cell are plotted and the absorptivity is calculated from the S-parameters.

## Additional Information

**How to cite this article**: Lee, D. *et al.* Incident Angle- and Polarization-Insensitive Metamaterial Absorber using Circular Sectors. *Sci. Rep.* **6**, 27155; doi: 10.1038/srep27155 (2016).
